# Polystyrene nanoplastics potentiate cadmium-induced pyroptosis and apoptosis in primary duck hepatocytes: differential responses to N-acetylcysteine

**DOI:** 10.3389/fvets.2026.1946639

**Published:** 2026-09-30

**Authors:** Zhixuan Chen, Yan Chen, Zhili Yu, Qingqing Xu, Qihang Li, Yonggang Ma, Xishuai Tong, Haibo Jin, Wei Liu, Hongyan Zhao, Jianhong Gu, Yan Yuan, Jianchun Bian, Zongping Liu, Hui Zou

**Affiliations:** 1 College of Veterinary Medicine, Yangzhou University, Yangzhou, Jiangsu, China; 2 Jiangsu Co-innovation Center for Prevention and Control of Important Animal Infectious Diseases and Zoonoses, Yangzhou University, Yangzhou, Jiangsu, China; 3 School of Public Health, Yangzhou University, Yangzhou, Jiangsu, China; 4 Research Facility Sharing Center, Yangzhou University, Yangzhou, Jiangsu, China

**Keywords:** apoptosis, cadmium, polystyrene nanoplastics, primary duck embryo hepatocytes, pyroptosis

## Abstract

**Introduction:**

Nanoplastics (NPs) can act as carriers for heavy metals, and co-exposure to NPs and co-pollutants may produce greater toxicity than single exposure. However, the combined toxicity of nanoplastics and heavy metals in poultry liver cells remains poorly understood.

**Methods:**

Primary duck embryonic hepatocytes (PDEH) were used as an *in vitro* model. Cells were exposed to polystyrene nanoplastics (PS-NPs, 80 nm, 10 μg/mL) and/or cadmium chloride (CdCl2, 4 μg/mL) for 12 h. Pyroptosis- and apoptosis-associated endpoints were assessed by brightfield microscopy, PI staining, SEM, flow cytometry, qRT-PCR, and Western blotting. The antioxidant N-acetylcysteine (NAC, 5 μg/mL) was used to evaluate the responses of these pathways.

**Results:**

Co-exposure to PS-NPs and Cd potentiated cadmium-induced pyroptosis-associated morphological changes, including cell swelling, rounding, membrane blebbing, and surface pore formation. Mechanistically, co-exposure activated the NLRP3/Caspase-1/GSDMD signaling axis, as indicated by increased NLRP3, cleaved Caspase-1 (P20/P10), GSDMD-NT, and intracellular IL-1β maturation. Co-exposure also exacerbated apoptosis-associated changes, including an increased Bax/Bcl-2 ratio and a higher Annexin V-positive cell population. NAC effectively reversed the apoptosis-associated endpoints but did not significantly alter the selected pyroptosis-associated markers under the present *in vitro* exposure conditions.

**Discussion:**

These findings suggest that PS-NPs potentiate Cd-induced hepatotoxicity in primary duck hepatocytes, with concurrent activation of pyroptosis- and apoptosis-associated signaling. The differential responses to NAC indicate that antioxidant intervention may preferentially attenuate apoptosis-associated injury under the present *in vitro* exposure conditions, while pyroptosis-associated signaling may require alternative targeted interventions. These results provide new insights into the combined toxicity of nanoplastics and heavy metals in avian hepatocytes.

## 1 Introduction

Since the 20th century, global plastic production has skyrocketed, yet its persistence in the natural environment has led to widespread pollution (1). Under prolonged physical abrasion, ultraviolet radiation, and microbial degradation, large plastic debris fragments into microplastics (MPs, < 5 mm) and further into nanoplastics (NPs, < 1 μm) (2). Due to their incredibly small size, immense specific surface area, and high hydrophobicity, NPs are not only capable of widespread migration across aquatic, terrestrial, and atmospheric environments but also readily traverse the intestinal epithelial barrier to enter the systemic circulation (3, 4). Recent studies have identified the presence of NPs in human blood, lung tissues, and even placentas, highlighting a threat to human health far more severe than previously anticipated (4–7). In the livestock industry, NPs can enter poultry through contaminated feed, drinking water, and even atmospheric deposition, leading to their bioaccumulation in metabolic target organs such as the liver and kidneys (8, 9).

Notably, the high specific surface area and surface charge characteristics of NPs enable them to act as carriers for various environmental toxicants (10). Trojan horse effect allows NPs to strongly adsorb heavy metals and organic pollutants, significantly altering their bioavailability and provoking combined toxicity that far exceeds the effects of single pollutant exposure (11). Cadmium (Cd) is a widely distributed industrial heavy metal pollutant, classified as a Group I carcinogen by the World Health Organization (12). Owing to its prolonged biological half-life of 20 to 30 years, Cd is rapidly absorbed and heavily accumulates in hepatic and renal tissues upon ingestion (12). In poultry farming, chronic dietary or waterborne intake of Cd not only induces hepatorenal injury and significantly reduces laying performance and weight gain in broilers but also leads to excessive Cd residues in animal-derived products (such as poultry meat and eggs), thereby jeopardizing the health of end consumers through the food chain (13, 14). Hepatotoxicity induced by such pollutants severely impacts poultry production economics and poses potential food safety risks for human consumption. Recently, emerging evidence has highlighted the Trojan horse effect of nanoplastics and the comparative toxicity among different nanoplastic types (e.g., polystyrene vs. polyethylene) in agricultural and livestock systems.

Extensive evidence indicates that biological damage induced by environmental pollutants is intimately linked with the aberrant activation of programmed cell death (PCD) (15, 16). Among these, pyroptosis is a recently identified pro-inflammatory form of cell death. In the canonical pyroptotic signaling cascade, external stimuli (such as heavy metal exposure or physical insult) first activate the intracellular NLRP3 inflammasome complex, which subsequently recruits and cleaves pro-Caspase-1 into its active form. Activated Caspase-1 not only cleaves full-length Gasdermin D (GSDMD) to release the N-terminal pore-forming domain (GSDMD-NT) but also promotes the maturation and release of the pro-inflammatory cytokines IL-1β and IL-18, triggering a robust inflammatory cascade (17). GSDMD-NT subsequently oligomerizes on the plasma membrane to form pores, leading to ion imbalance, cellular swelling, and eventual rupture (18). On the other hand, apoptosis is a highly ordered, non-inflammatory form of PCD primarily regulated by the intrinsic mitochondrial pathway. It is characterized by the upregulation of pro-apoptotic proteins and the downregulation of anti-apoptotic proteins (19). An elevated Bax/Bcl-2 ratio induces mitochondrial membrane potential collapse, releasing cytochrome c to activate downstream caspase cascades, ultimately leading to cell shrinkage, DNA fragmentation, and apoptotic body formation (20). Although previous studies have confirmed that Cd or PS-NPs alone can induce hepatocyte apoptosis or pyroptosis, how combined exposure to both pollutants concurrently influences and differentially regulates these two distinct death pathways in avian hepatocytes remains mechanistically underexplored (21–23).

The massive outburst of reactive oxygen species (ROS) is widely accepted as the core upstream event underlying Cd and NP-induced cytotoxicity. Cd disrupts mitochondrial function, leading to excessive electron leakage from the electron transport chain and subsequent ROS bursts, while intracellular NPs may further aggravate this process by inducing mitochondrial membrane potential decline (24–26). As a converging point for multiple cell death signals, oxidative stress represents a potential therapeutic target. N-acetylcysteine (NAC), a classical antioxidant and precursor of glutathione, effectively scavenges intracellular and extracellular free radicals and serves as an indispensable pharmacological tool for validating the mediating role of oxidative stress (27, 28). Consequently, employing NAC as an intervention tool not only helps uncover the role of oxidative stress in combined exposure but also elucidates the differential susceptibility of apoptosis and pyroptosis to ROS scavenging.

Based on the aforementioned background, this study employed primary duck embryo hepatocytes (PDEH) as an *in vitro* model to systematically investigate the specific modalities and molecular mechanisms of hepatocyte death induced by co-exposure to 80 nm PS-NPs and Cd. Our investigations focused not only on the expression changes in the canonical NLRP3/Caspase-1/GSDMD pyroptotic pathway and the Bax/Bcl-2-mediated mitochondrial apoptotic pathway, but also on the differential regulatory effects of antioxidant intervention on pyroptosis vs. apoptosis through NAC co-treatment experiments. This research aims to bridge the existing gap in the toxicological understanding of combined NPs and heavy metal pollutants in avian hepatocytes, thereby providing a robust theoretical basis for the formulation of prevention strategies against combined pollution in the poultry industry and the development of targeted therapeutic interventions against specific cell death pathways.

## 2 Materials and methods

### 2.1 Chemicals and reagents

Table 1 lists the antibodies utilized in this study. Table 2 lists the drugs and kits utilized in this study. The plain and the green-fluorescent PS-NPs of size 80 nm were purchased from BaseLine (Tianjin, China). The size of the particles was characterized by Scanning Electron Microscope (SEM) and Malvern nano-particle size analyzer (Malvern Instruments, UK). Analytical grade chemicals and reagents were bought locally.

**Table 1:** Antibodies used in present study.

Antibody	Catalog number	Company
Bax	T40051	Abmart
Bcl-2	T40056	Abmart
NLRP3	30109-1-AP	Proteintech
IL-1β	16806-1-AP	Proteintech
Caspase-1	22915-1-AP	Proteintech
GSDME	66387-1-Ig	Proteintech
GSDMD	WL05686	Wanleibio
GSDMD N-terminal	WL05411	Wanleibio
GAPDH	60004-1-Ig	Proteintech
β-Actin	66009-1-Ig	Proteintech
Goat anti-rabbit	7074	Cell Signaling Technology
Horse anti-mouse	7076	Cell Signaling Technology

**Table 2:** Drug and kit used in present study.

Name	Catalog no.	Company
CdCl_2_	202908	Sigma-Aldrich
PS-NPs	6-1-0008	BaseLine
BCA Kit	P0009	Beyotime
Annexin V-FITC Apoptosis Detection Kit	C1062M	Beyotime
Collagenase IV	BS165-100mg	Biosharp
1 × PBS buffer	BL310A	Biosharp
RIPA Lysis Buffer	WB3100	NCM Biotech
Glutaraldehyde fixative (2.5%)	BL-G014	Senbeijia

### 2.2 PDEH cell culture

PDEH were isolated from 15–18-day-old duck embryos (Gaoyou Duck Breeding Base, Yangzhou, China) by type IV collagenase digestion as described previously (29). Excised liver samples were dissected into small fragments and enzymatically dissociated with collagenase at 37 °C for 1 h. The cell suspension was strained through a 70-μm mesh and centrifuged at 800 rpm for 5 min to collect the cell pellet. Isolated cells were seeded in Williams' Medium E (Sigma-Aldrich) supplemented with 10% fetal bovine serum, 100 U/mL penicillin, and 100 μg/mL streptomycin, and incubated at 37 °C under 5% CO_2_. For the preparation of PS-NPs, the stock solution was sonicated in an ultrasonic bath (40 kHz) for 5 min to ensure adequate dispersion. The desired working concentration (10 μg/mL) was then freshly prepared by diluting the sonicated stock solution with the cell culture medium immediately prior to the exposure experiments. The concentration of NAC (5 μg/mL) was selected based on preliminary CCK-8 cytotoxicity assays and previous literature, which showed no significant reduction in cell viability at this dose (25). For the NAC intervention, cells were pretreated with serum-free medium containing 5 μg/mL NAC for 1 h. After this pretreatment, the medium was replaced with fresh serum-free medium containing Cd (4 μg/mL) alone, PS-NPs (10 μg/mL) alone, or a combination of Cd and PS-NPs, in the continuous presence of NAC (5 μg/mL). The cells were then incubated for an additional 12 h (25).

### 2.3 Flow cytometry (FCM)

PDEH cells were subjected to a toxicity treatment, followed by collection through centrifugation. The cells were then stained with a fluorescent probe and analyzed via flow cytometry (LSRFortessa, BD Biosciences, USA). Briefly, cells were inoculated into six-well plates, exposed with Cd and NPs for 12 h, then loaded with probes. The plates were then washed three times with PBS in order to remove any residual probes. Cells were co-incubated with EDTA-free trypsin for 40 s. Following this, cells were collected by means of centrifugation, resuspended in Phosphate Buffered Saline (PBS) and prepared as single-cell suspensions by sieving through a 200-mesh filter. For the flow cytometry analysis of apoptosis, cells were first gated on the forward scatter (FSC) vs. side scatter (SSC) dot plot to exclude cellular debris. Doublets were then excluded using FSC-H vs. FSC-A gating. The single-cell population was subsequently analyzed on a bivariate dot plot of Annexin V-FITC (X-axis) vs. PI fluorescence (Y-axis). Quadrants were set based on unstained, FITC single-stained, and PI single-stained controls. Cells in the lower-right quadrant (Annexin V+/PI–) were defined as early apoptotic, and those in the upper-right quadrant (Annexin V+/PI+) were defined as late apoptotic/necrotic. The total apoptotic rate was calculated as the sum of early and late apoptotic cells.

### 2.4 Scanning electron microscopy (SEM)

PDEH cells were placed onto glass coverslips in 24-well plates. Upon reaching 70% confluency, the culture medium was exchanged for fresh medium containing Cd alone, PS NPs alone, or a mixture of both, and the cells were incubated for an additional 12 h. For SEM examination, the specimens were fixed in 2.5% glutaraldehyde at ambient temperature for 2 h, followed by graded ethanol dehydration (15 min per step), air-drying, gold-coating by sputtering, and finally observation under a GeminiSEM 300 scanning electron microscope (Carl Zeiss, Germany).

### 2.5 Malvern nano-particle size analyzer

The hydrodynamic diameter and polydispersity index (PDI) of the 80 nm PS-NPs were determined by dynamic light scattering (DLS) on a Malvern nano-particle size analyzer (Malvern Instruments, Malvern, UK). For measurement, the NPs were suspended in ultrapure water at 10 μg/mL, ultrasonicated for 5 min, and loaded into a cuvette. DLS data were acquired at 25 °C with a 173° backscatter angle. Each sample was read in triplicate, and the results are expressed as mean ± standard deviation (SD). The Z-average size and PDI values were extracted from cumulants analysis. The summarized Z-average diameter and PDI data are provided in Table 3.

**Table 3:** Z-average size and PDI of 80 nm PS-NPs in ultrapure water.

Sample	Z-average (d.nm)	PDI
PS-NPs-1	80.50	0.145
PS-NPs-2	78.85	0.268
PS-NPs-3	81.61	0.171

### 2.6 Live-cell imaging and PI staining

PDEH cells were seeded in 24-well plate at a density of 2.5 × 10^5^ cells per well. Then, add serum-free medium containing Cd and/or PS-NPs to replace the complete medium and incubate for 12 h. Cell morphology was monitored under brightfield microscopy. For PI staining, cells were incubated with 1 μg/mL propidium iodide for 10 min at 37 °C. Fluorescence images were captured under identical magnification. PI-positive cells were counted and normalized to total cell number obtained from the corresponding brightfield images (AXIO observer 7, Carl Zeiss, Germany).

### 2.7 Western boltting

To obtain whole-cell lysates, PDEH cells were disrupted in RIPA buffer that contained a protein phosphatase inhibitor cocktail (100 ×, P1260, Beijing, China). The suspension was sonicated and centrifuged, and the resulting supernatant was collected for protein quantification. Equal protein aliquots were fractionated by SDS-PAGE and transferred to PVDF membranes (IPVH00010, Millipore, Billerica, USA). Nonspecific binding was blocked with 5% non-fat milk for 2 h at 25 °C. The membranes were then incubated overnight at 4 °C with primary antibodies, washed, and exposed to HRP-conjugated secondary antibodies for 2 h at room temperature. Immunoreactive bands were visualized with a chemiluminescent reagent (WBKLS0500, Millipore) and the relative intensities were quantified using ImageJ and GraphPad Prism 9. Information regarding antibody dilutions and validation for duck proteins is provided in Table 1 and on the official websites of the respective antibody manufacturers.

### 2.8 qRT-PCR analysis

Total RNA was isolated from PDEH cells using TRIzol^®^ reagent (Life Technologies, CA, USA), and its concentration was determined with a NanoDrop™ 2000C spectrophotometer (Thermo Fisher Scientific). First-strand cDNA was then synthesized from equal amounts of RNA using a reverse transcription kit, following the supplier's recommended protocol. Quantitative real-time PCR was performed on an Applied Biosystems 7500 system (Life Technologies, CA, USA) with SYBR Green master mix (YEASEN, Shanghai, China). GAPDH served as the endogenous reference gene, and relative transcript levels were calculated via the 2^−Δ*ΔCt*^ method. All primer sequences used are provided in Table 4.

**Table 4:** Gene primers sequence used in present study.

Gene	Forward primer	Reverse prime
NLRP3	ACAGCTTCACACACCTGCAC	GTGAAATTCTGCACCCGATT
IL-1β	TGGCACTGGGCATCAAGGG	GGCTGTCCAGGCGGTAGAAG
ASC	CAGCATTCTGGATCGGCTCT	ATTTTCTCCTGCCTGATGCTT
IL-18	AACCCTCCATCGCTTCCTTCG	AGTTCATTCCGCTGCCAGATTTC
Caspase-1	TCGGTGCTGGTGTCTGACTCA	AGACGGTATCAGGTGTGGAGGA
GSDMA	GTGTGAACTCCTGCTCCGTGA	TGTTGCGTCTTCCGTAGTTGCT
GSDME	TGGGCATCAAGGGCTACA	CCTAAGAGCTGCAAGAAGTCAACC
NEK7	GATGGATTCGTGAGCGTGGACA	TCCGGGTTGATGCACATATTA
Bax	CACCAAGGAGAATGCCTACGAT	GGCCCCAGTTGAAGGCTCCGTCC
Bcl-2	ATGTCGCCCTGGATTTCG	TGCAAGCTCCCACCAGAACCA
GAPDH	GCCACACAGAAGACAGTGGA	AGGTCCACGACAGAGACGTT

### 2.9 Statistical analysis

All statistical calculations were carried out using GraphPad Prism 9. For multi-group comparisons (≥3 groups), one-way analysis of variance (ANOVA) followed by Tukey's Honest Significant Difference (HSD) *post-hoc* test was performed. For direct two-group comparisons, Student's unpaired t-test was used. Data were obtained from three independent biological replicates (*n* = 3). Each replicate represents an independent primary hepatocyte isolation and culture preparation, for which the livers from four duck embryos were pooled to obtain a sufficient cell yield for the subsequent experimental treatments. The results are presented as the mean ± SD. A significance threshold of *p* < 0.05 was adopted. All statistical symbols (^*^, ^**^, #, ##, ns) denote significance differences defined in the figure legends.

## 3 Results

### 3.1 Characterization of 80 nm PS-NPs and their interaction with PDEH cells

The physical properties of the 80 nm PS-NPs were comprehensively characterized. SEM revealed that the nanoparticles exhibited a well-defined spherical morphology with a relatively uniform primary size distribution when dispersed in ultrapure water (Figure 1A). DLS analysis indicated a mean hydrodynamic diameter of approximately 80.50 ± 1.63 nm and a narrow PDI of 0.145 to 0.268 (Figure 1C and Table 3). This low PDI confirms the high stability and monodispersity of the nanoparticle suspensions. To assess cellular uptake, PDEH cells were incubated with fluorescently labeled FPS-NPs (green). As shown in Figure 1B, after incubation, strong and punctate green fluorescence was distributed within the cytoplasm, predominantly accumulating in the perinuclear region of PDEH cells. When these PS-NPs were dispersed in the cell culture medium used for the *in vitro* exposure, microscopic observations indicated that the nanoparticles formed distinct aggregates/agglomerates, which is a common phenomenon for nanomaterials in protein-rich physiological media and more accurately reflects their actual state during cellular exposure. Cellular identity was supported by Glycogen Periodic Acid–Schiff (PAS) staining, which produced strong magenta-purple staining within the cytoplasm of the majority of cells, indicating abundant glycogen storage characteristic of this cell type (Figure 1E). The high purity of the primary hepatocyte population was routinely verified based on our established cell isolation protocol and morphological characteristics (29). Furthermore, time-course brightfield imaging was performed from 0 to 36 h post-seeding. Cells adhered to the surface within 12 h and progressively proliferated, forming a confluent monolayer with typical polygonal morphology and distinct cell-cell contacts by 36 h (Figure 1D). This verifies that PDEH cells maintain normal proliferation and adhesion characteristics under standard culture conditions.

**Figure 1:**
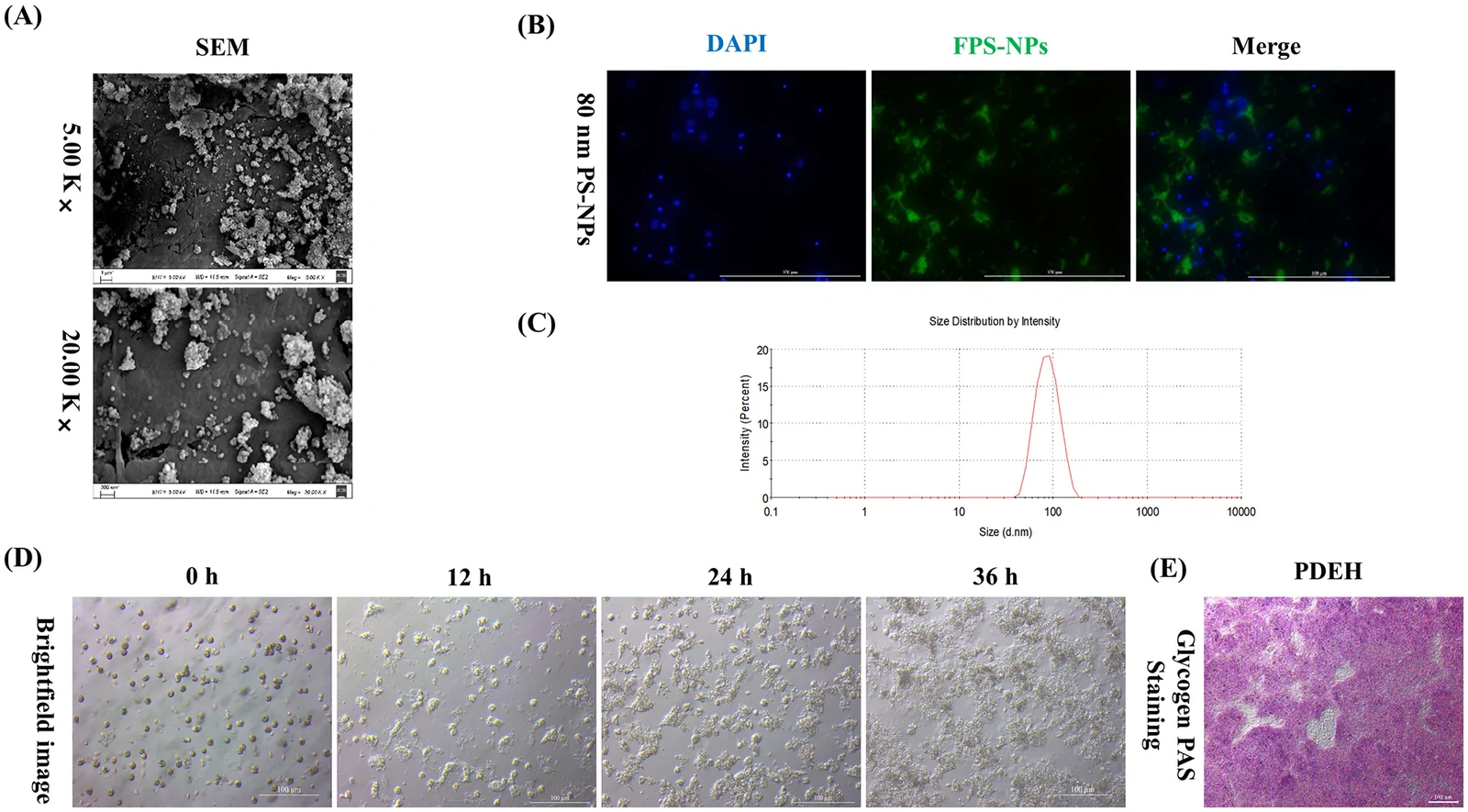
Characterization of 80 nm PS-NPs and their interaction with PDEH cells. PDEH cells were exposed to Cd (4 μg/mL) or PS-NPs (10 μg/mL) alone or in combination for 12 h. **(A)** Scanning electron microscopy (SEM) image of 80 nm PS-NPs (Scale bars = 1 μm and 300 nm). **(B)** Representative fluorescence microscopy images of PDEH cells after exposure to fluorescently labeled PS-NPs (green). Nuclei were counterstained with DAPI (blue). The merged image shows intracellular localization of PS-NPs. **(C)** Hydrodynamic size distribution of 80 nm PS-NPs measured by dynamic light scattering (DLS). **(D)** Time-course brightfield images of PDEH cells recorded at 0, 12, 24, and 36 h post-seeding. **(E)** Periodic acid–Schiff (PAS) staining of PDEH cells (Scale bars = 100 μm). *n* = 3.

### 3.2 PS-NPs exacerbate cadmium-induced pyroptosis in PDEH cells

Next, this study evaluated the impact of PS-NPs and Cd on cell morphology. As shown in the brightfield images (Figure 2A), cells in the control group exhibited a healthy, spread-out morphology with clear edges. While cells treated with Cd alone or NPs alone displayed only mild morphological alterations such as slight rounding, the combined exposure (PS-NPs+Cd) induced severe morphological changes. These included pronounced cell shrinkage, a complete loss of cell-cell contact, and extensive membrane blebbing—features entirely consistent with pyroptosis. To quantify membrane integrity damage, PI staining was performed. As shown in Figure 2B, the number of PI-positive cells (red fluorescence) was substantially higher in the PS-NPs+Cd group compared to the control group or individual treatment groups. SEM further corroborated these observations at the ultrastructural level. While control cells exhibited intact microvilli and a relatively smooth cell surface with well-preserved filopodia, cells in the PS-NPs+Cd group showed extensive membrane rupture, the formation of multiple distinct pores (ranging from 0.5 to 1.5 μm in diameter), and severe disintegration of the cell surface, leaving behind a “spongy” appearance (Figure 2C).

**Figure 2:**
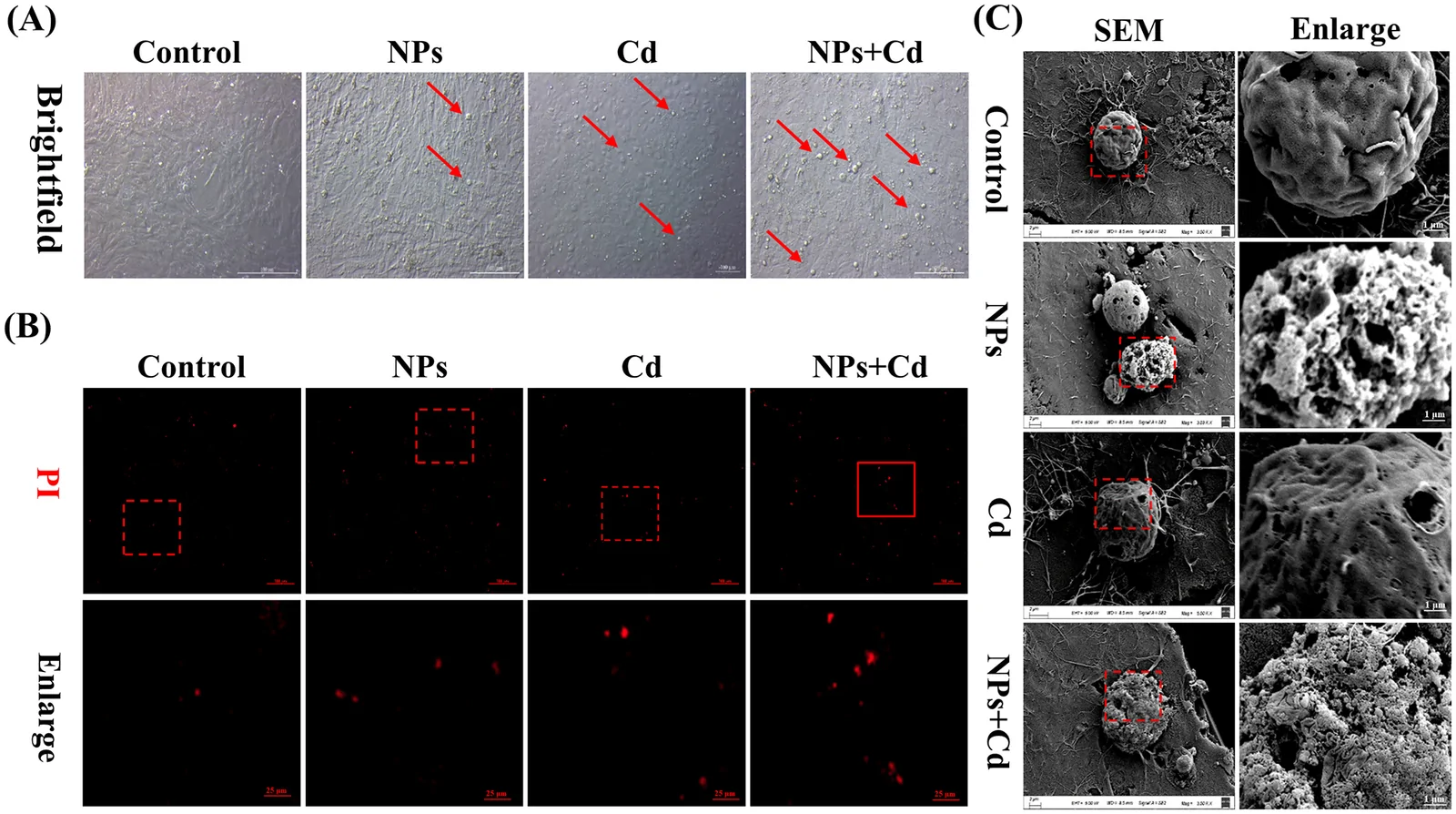
PS-NPs exacerbate cadmium-induced pyroptosis in PDEH cells. PDEH cells were exposed to Cd (4 μg/mL) or PS-NPs (10 μg/mL) alone or in combination for 12 h. **(A)** Brightfield microscopy images of PDEH cells following different treatments. The red arrows indicate pyroptotic-like cells (Scale bars = 100 μm). **(B)** Representative images of propidium iodide (PI) staining (red fluorescence) in PDEH cells. PI-positive cells indicate loss of plasma membrane integrity (Scale bars = 100 μm and 25 μm). **(C)** Scanning electron microscopy (SEM) images of PDEH cells showing surface ultrastructure. Enlarged views (right panels) provide higher magnification details of membrane damage (Scale bars = 2 μm and 1 μm). *n* = 3.

### 3.3 Combined Cd and PS-NPs upregulate pyroptosis-related genes and proteins irrespective of NAC

To elucidate the molecular mechanism driving this exacerbated cell death, the expression of key pyroptosis-related genes and proteins was analyzed. Quantitative RT-PCR showed that exposure to PS-NPs+Cd significantly upregulated the mRNA levels of *GSDMA, GSDME, NEK7, Caspase-1, NLRP3, ASC*, and *IL-1*β compared to the control group or individual treatments (*p* < 0.01). For instance, *GSDME* mRNA levels increased by approximately 2.1-fold compared to the control group (Figures 3A, B). Consistent with the gene expression data, Western blot analysis confirmed that the protein levels of NLRP3, IL-1β, GSDME, full-length Caspase-1, its active cleaved forms (P20 and P10), GSDMD, and the pore-forming fragment GSDMD-NT were all significantly elevated in the co-exposure group (*p* < 0.01; Figures 3C–E). To determine whether oxidative stress mediated this process, we introduced the antioxidant NAC (5 μg/mL). Strikingly, the addition of NAC did not significantly attenuate the upregulation of any of these pyroptosis-related genes or proteins (*p* > 0.05). This indicates that the PS-NPs+Cd-induced pyroptosis is largely independent of NAC-sensitive oxidative stress pathways in this model. The above results suggest that PS-NPs can exacerbate Cd-mediated pyroptosis through the canonical NLRP3/Caspase-1/GSDMD pathway, and this process is resistant to NAC intervention.

**Figure 3:**
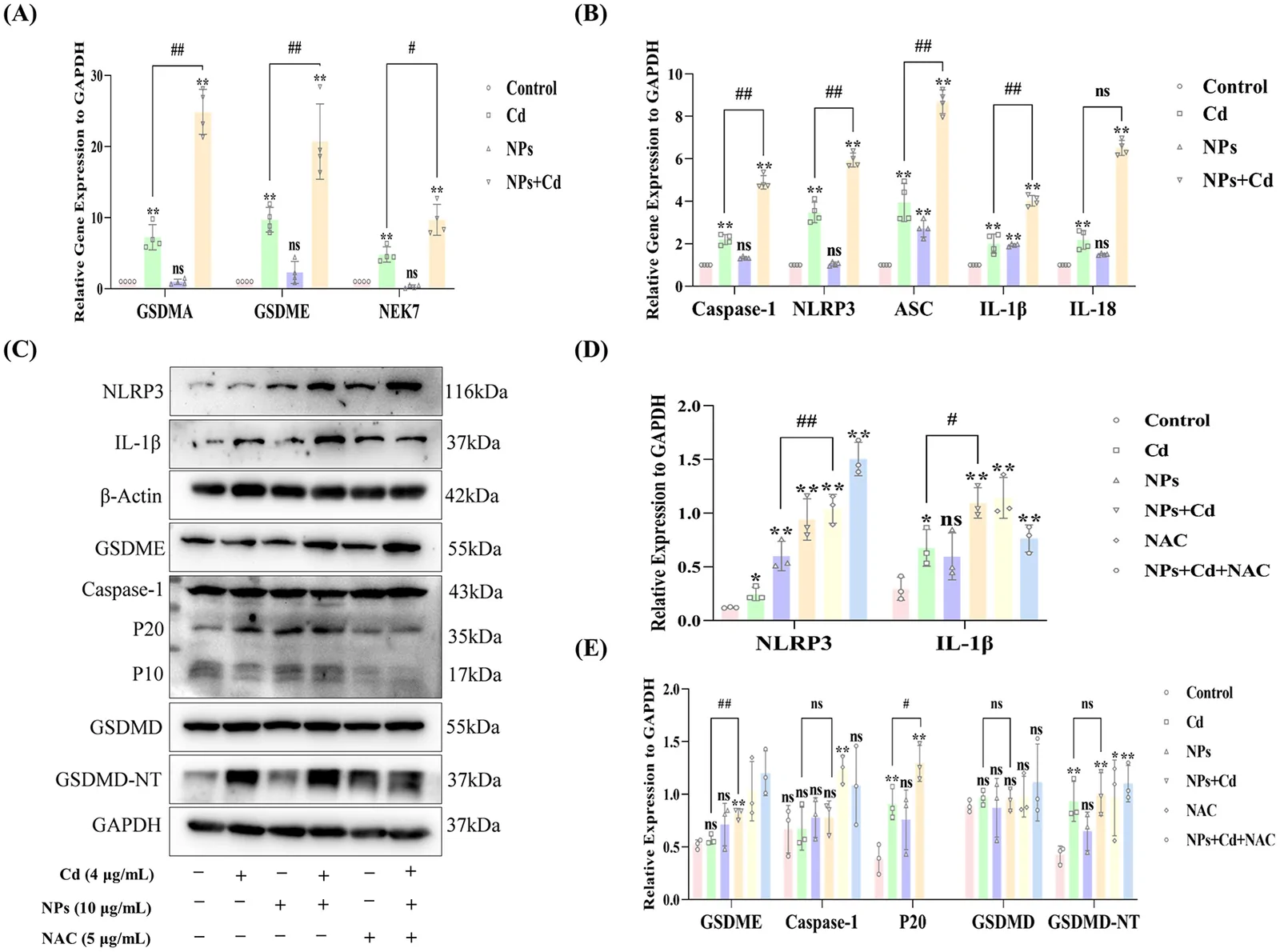
Combined Cd and PS-NPs upregulate pyroptosis-related genes and proteins irrespective of NAC. PDEH cells were exposed to Cd (4 μg/mL), PS-NPs (10 μg/mL), and/or NAC (5 μg/mL) for 12 h. **(A, B)** Quantitative RT-PCR analysis of the relative mRNA expression levels of pyroptosis-related genes (*GSDMA, GSDME, NEK7, Caspase-1, NLRP3, ASC, IL-1*β, and *IL-18*) in cells, using *GAPDH* as the internal control. **(C)** Representative Western blotting images showing the protein expression levels of NLRP3, IL-1β, GSDME, Caspase-1 (and its cleavage products P20 and P10), and GSDMD (and its N-terminal fragment GSDMD-NT). β-Actin and GAPDH were used as loading controls. **(D, E)** Quantitative analysis of **(C)** *n* = 3. Statistical significance was determined by one-way analysis of variance (ANOVA) followed by Tukey's Honest Significant Difference (HSD) *post-hoc* test for multiple group comparisons. Data are presented as mean ± SD. *p < 0.05; **p < 0.01; ^#^*p* < 0.05; ^##^*p* < 0.01; ns means no significant.

### 3.4 Combined Cd and PS-NPs induce apoptosis in PDEH cells, an effect reversed by NAC

In addition to pyroptosis, this study investigated whether the combined exposure induced cell apoptosis. In the PS-NPs+Cd co-treatment group, the transcript and protein levels of the pro-apoptotic factor Bax were significantly elevated relative to the control, whereas those of the anti-apoptotic protein Bcl-2 were substantially reduced (*p* < 0.01, Figure 4). Specifically, Bax protein showed an approximate 2-fold increase, while Bcl-2 dropped to about 40% of the control value, yielding a markedly higher Bax/Bcl-2 ratio—a characteristic indicator of the mitochondrial (intrinsic) apoptotic cascade (Figures 4A–C). To directly quantify the apoptotic population, cells were stained with Annexin V-FITC/PI and analyzed by flow cytometry. As illustrated in Figures 4D, E, the PS-NPs+Cd group exhibited a significantly greater proportion of apoptotic cells (Annexin V-positive) than the control, with total apoptosis reaching 35.2 ± 2.1% (comprising 12.3% early and 22.9% late apoptosis) vs. 16.5 ± 1.4% (6.0% early and 10.5% late) in the control (*p* < 0.01). These findings suggest that the combined exposure induces phenotypic changes consistent with the concurrent activation of both apoptotic and pyroptotic pathways.

**Figure 4:**
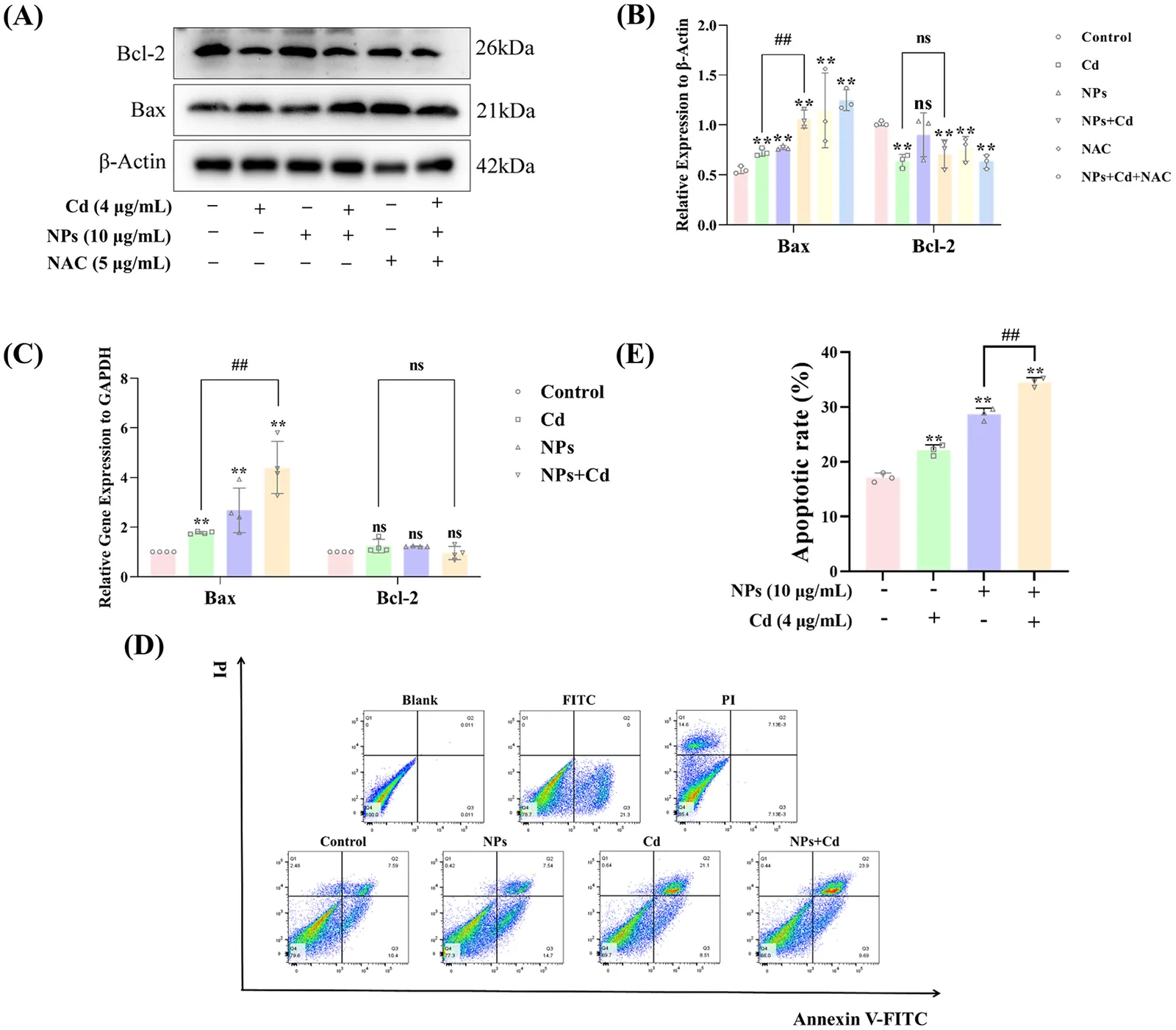
Combined Cd and PS-NPs induce apoptosis in PDEH cells, an effect reversed by NAC. PDEH cells were exposed to Cd (4 μg/mL), PS-NPs (10 μg/mL), and/or NAC (5 μg/mL) for 12 h. **(A)** Representative Western blotting images showing the protein expression levels of Bax and Bcl-2. β-Actin was used as the loading control. **(B)** Quantitative analysis of the relative expression levels of Bax and Bcl-2 proteins normalized to β-Actin. **(C)** Relative mRNA expression levels of *Bax* and *Bcl-2* determined by quantitative RT-PCR, using *GAPDH* as the internal control. **(D)** Representative flow cytometry dot plots of cell apoptosis stained with Annexin V-FITC and PI. **(E)** Quantification of the percentage of apoptotic cells (apoptotic rate) based on the flow cytometry analysis in **(D)**. Statistical significance was determined by one-way analysis of variance (ANOVA) followed by Tukey's Honest Significant Difference (HSD) *post-hoc* test for multiple group comparisons. Data are presented as mean ± SD. *p < 0.05; **p < 0.01; ^##^*p* < 0.01; ns means no significant; *n* = 3.

### 3.5 NAC reverses apoptosis but not pyroptosis induced by combined NPs and Cd in PDEH cells

Given that NAC failed to inhibit pyroptosis markers (Figure 3), this study sought to further examine its specific effect on the apoptotic component and overall cell fate. Brightfield microscopy showed that the addition of NAC to the PS-NPs+Cd group partially restored normal cell morphology compared to the severe damage seen in the PS-NPs+Cd alone group. Many cells regained a more flattened, spread-out morphology, though some rounded cells remained (Figure 5A). Importantly, NAC treatment did not significantly alleviate the red fluorescence from PI staining compared to the PS-NPs+Cd group. This persistent red fluorescence suggests that while apoptosis was rescued, membrane damage from pyroptosis was still ongoing (Figure 5B). Flow cytometry analysis revealed that the addition of NAC to the NPs+Cd group significantly reduced the total apoptotic rate from approximately 33.1 ± 1.8% down to 22.4 ± 1.5% (*p* < 0.01; Figures 5C, D). Specifically, early apoptosis decreased from 11.0% to 6.5%, and late apoptosis decreased from 22.1% to 15.9%. Collectively, these data demonstrate that NAC effectively reversed the apoptosis-associated endpoints but did not significantly alter the selected pyroptosis-associated markers under the present *in vitro* exposure conditions.

**Figure 5:**
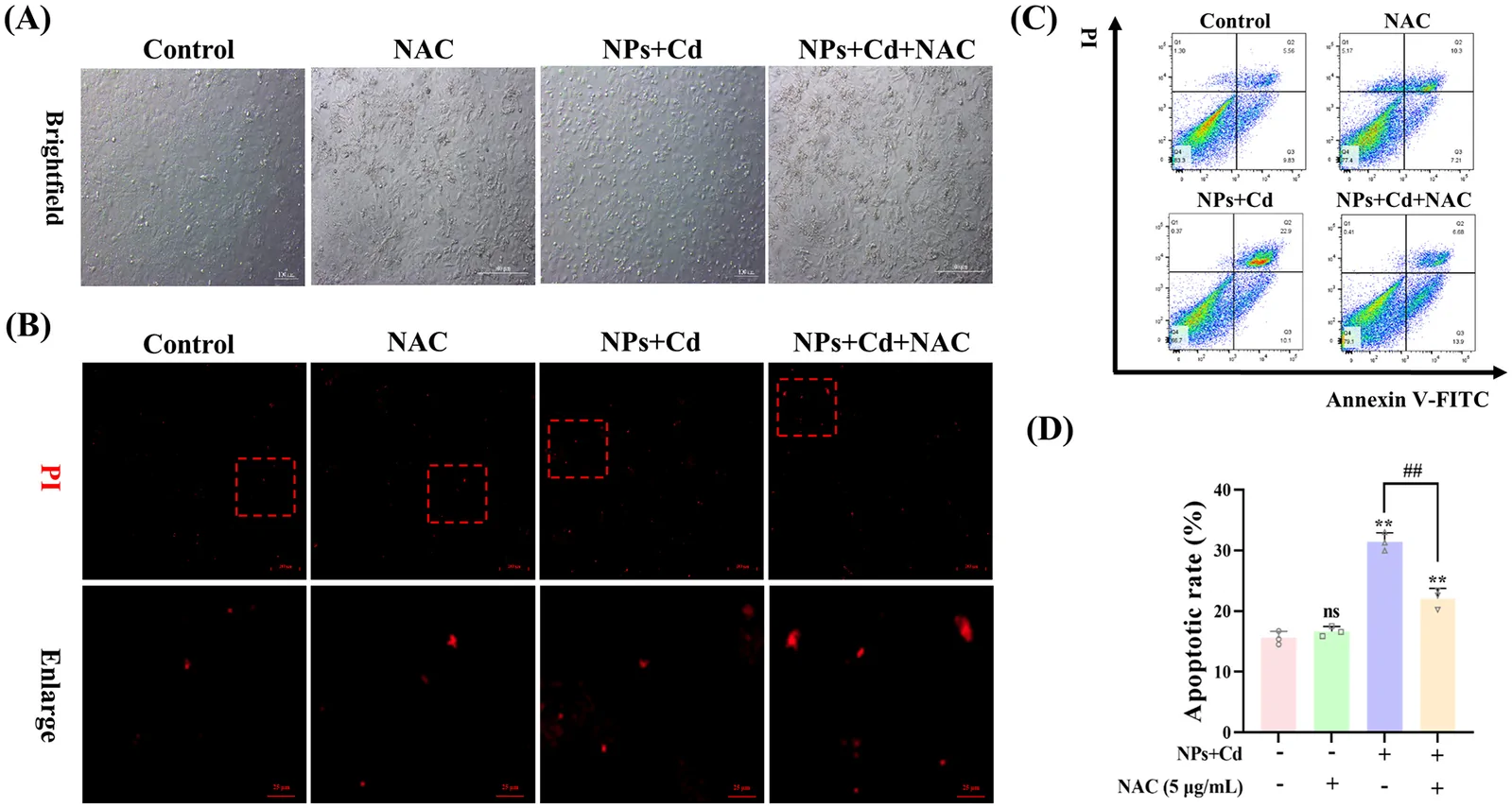
NAC reverses apoptosis but not pyroptosis induced by combined NPs and Cd in PDEH cells. PDEH cells were exposed to Cd (4 μg/mL), PS-NPs (10 μg/mL), and/or NAC (5 μg/mL) for 12 h. **(A)** Brightfield microscopy images showing cellular morphological changes across different treatment groups (Scale bars = 100 μm). **(B)** Representative images of PI staining (red) for dead cells, with red dashed boxes indicating the enlarged areas (Scale bars = 100 μm and 25 μm). **(C)** Flow cytometry analysis of cell apoptosis quantified by Annexin V-FITC and PI double staining. **(D)** Statistical analysis of the total apoptotic rate (%) determined from panel. Statistical significance was determined by one-way analysis of variance (ANOVA) followed by Tukey's Honest Significant Difference (HSD) *post-hoc* test for multiple group comparisons. Data are presented as mean ± SD. *p < 0.05; **p < 0.01; ^##^*p* < 0.01; ns means no significant; *n* = 3.

## 4 Discussion

Plastic pollution has evolved into a globally recognized environmental and public health crisis. With the continuous detection of MPs and NPs in human blood, placentas, and various biological tissues, their potential health risks extend far beyond ecological concerns and directly threaten the safety of the food chain (4, 6, 8). Using PDEH as an *in vitro* model, this study systematically elucidated, for the first time, the induction of two major programmed cell death pathways—pyroptosis and apoptosis—in poultry hepatocytes following co-exposure to 80 nm PS-NPs and the heavy metal Cd.

First, our fluorescence imaging and SEM results demonstrated that 80 nm PS-NPs can efficiently penetrate the PDEH cell membrane and accumulate in the perinuclear region, which is consistent with the high penetrability and intracellular distribution characteristics of NPs due to their extremely small particle size. More critically, the addition of Cd significantly exacerbated the intracellular accumulation of PS-NPs. These findings are consistent with the possibility that Cd alters PS-NP cellular uptake: owing to their large specific surface area and surface charge, NPs can not only act as physical carriers to adsorb Cd ions but also disrupt the stability of the lipid bilayer of the cell membrane, increasing passive membrane permeability (30, 31). This creates a vicious cycle of Cd-induced pore formation and NP influx, ultimately leading to cellular damage that far exceeds that caused by single pollutant exposure (32). This combined uptake mechanism explains, from a physicochemical perspective, why the cytotoxicity of co-exposure exhibits such a pronounced amplification effect. Based on cellular morphology, molecular biology, and flow cytometry results, the co-exposure group exhibited highly typical pathological features in PDEH cells. SEM clearly captured cellular swelling and rounding, the presence of membrane pores approximately 0.5–1.5 μm in diameter, and spongy disintegration of the membrane surface. PI staining further confirmed the irreversible destruction of membrane integrity. At the molecular level, co-exposure significantly activated the canonical NLRP3 inflammasome and its downstream signaling axis, including Caspase-1 activation, the cleavage of GSDMD to produce the pore-forming fragment GSDMD-NT, and the maturation and release of the pro-inflammatory cytokine IL-1β. This indicates the activation of the canonical pyroptotic signaling pathway. Concurrently, under the same exposure conditions, the pro-apoptotic protein Bax was significantly upregulated, while the anti-apoptotic protein Bcl-2 was strongly suppressed, leading to a sharp increase in the Bax/Bcl-2 ratio, and flow cytometry confirmed a dramatic rise in the apoptosis rate. This evidence indicates that co-exposure to PS-NPs and Cd does not merely activate a single cell death pathway, but rather simultaneously ignites two distinct death signals—pyroptosis and apoptosis—in the same spatiotemporal context. The combined action of these two death modalities drastically reduces hepatocyte survival.

To elucidate the mediating role of oxidative stress in combined toxicity, we introduced the classical free radical scavenger NAC. Surprisingly and encouragingly, the effects of NAC treatment exhibited a stark dichotomy: Following NAC administration, the Bax/Bcl-2 ratio was significantly reduced, the apoptosis rate measured by flow cytometry notably decreased, and the majority of damaged cells regained a relatively flattened and spread-out morphology. This fully demonstrates that the mitochondrial apoptotic pathway is highly dependent on the accumulation of intracellular ROS. Prior to cytochrome c release and caspase cascade activation, NAC possesses a clear intervention window (33). Antioxidants can effectively repair mitochondrial damage caused by ROS and block the further transduction of apoptotic signals (34, 35). In stark contrast to apoptosis, the addition of NAC failed to induce any significant downregulation of protein expression for NLRP3, Caspase-1, GSDMD, and GSDMD-NT. Furthermore, the number of PI-positive cells (cells with compromised plasma membranes) remained significantly high. This implies that the canonical pyroptotic pathway strongly resists antioxidant intervention by NAC, indicating that its activation is not absolutely dependent on ROS.

The failure of this antioxidant can be deeply interpreted from the following three aspects: First, apoptosis possesses a reversible amplification space prior to Caspase-3 activation (36). However, in the pyroptotic pathway, once Caspase-1 is activated and cleaves GSDMD, the resulting GSDMD-NT immediately oligomerizes on the cell membrane to form irreversible pores. The complete collapse of intracellular and extracellular ion gradients caused by these membrane pores cannot be repaired by later ROS scavenging (37). Second, as the core trigger for pyroptosis, the activation mechanism of the NLRP3 inflammasome extends far beyond the ROS pathway. Numerous studies have confirmed that intracellular potassium (K+) efflux, lysosomal rupture releasing cathepsin B, and the leakage of mitochondrial DNA (mtDNA) into the cytosol can all effectively activate the NLRP3 inflammasome through non-oxidative stress signals (38, 39). We acknowledge that these specific non-ROS pathways are plausible hypotheses based on existing literature, but they were not directly verified in the present study. In this study, PS-NPs, as rigid and non-degradable synthetic polymeric particles, may remain trapped in lysosomes, potentially leading to physical rupture of the lysosomal membrane. This mechanical stress can bypass ROS and directly activate pyroptosis, which is why NAC cannot block it (40, 41). Third, as a heavy metal, Cd not only induces ROS but also binds to protein sulfhydryl groups on the cell membrane, directly compromising membrane fluidity and forming pre-damage pores (42). These pre-existing microscopic pores open pathways for the subsequent passive influx of PS-NPs (43). This combined physicochemical attack, characterized by Cd-induced chemical perforation + NPs-induced mechanical disruption, pushes cellular damage beyond the realm of simple oxidative stress toward irreversible mechanical disintegration.

On the applied level for clinical and farming practices, this differential regulation holds significant implications. When facing the risk of combined exposure to nanoplastics and heavy metals, the simple use of broad-spectrum antioxidants such as NAC may only partially mitigate apoptotic injury but remain ineffective against pyroptosis. Therefore, when formulating intervention strategies, targeting the apoptotic pathway may offer more immediate efficacy than simply inhibiting pyroptosis. Intervening against pyroptosis may require targeting Caspase-1, GSDMD, or upstream non-ROS signals that activate the NLRP3 inflammasome. Nevertheless, this study has limitations. First, *in vitro* cell models cannot recapitulate the complex immune microenvironment, hepatic metabolic networks, and systemic physiological responses found *in vivo*. Second, the specific deep molecular switches accounting for NAC's inability to reverse pyroptosis warrant further investigation through *in vivo* experiments and protein structural analyses. Third, given that our fixed single-dose design precludes formal factorial analysis to distinguish additive from synergistic toxicity, our conclusions are strictly limited to describing the observed cellular and molecular responses under these specific exposure conditions, warranting future multi-dose studies for definitive interaction assessment. Therefore, future *in vivo* studies, such as duck feeding trials with environmentally relevant concentrations of PS-NPs and Cd, are essential to validate the hepatic pathological changes and differential responses to NAC observed in the present model. In future studies, the application of advanced bioimaging techniques, such as aggregation-induced emission (AIE) fluorescence probes, may serve as promising tools for the real-time quantification of nanoplastics and heavy metals in biological tissues (44, 45). Such approaches would help further validate the internalization and intracellular trafficking of these pollutants *in vivo*.

## 5 Conclusion

This study demonstrates that co-exposure to PS-NPs and cadmium induces hepatotoxicity in PDEH cells via concurrent pyroptosis and apoptosis. Mechanistically, the observed cell death features are associated with the activation of the canonical NLRP3/Caspase-1/GSDMD pathway, while apoptosis involves an elevated Bax/Bcl-2 ratio. Notably, NAC effectively reverses apoptosis but fails to mitigate pyroptosis, indicating that these two pathways exhibit differential responses to NAC under the tested conditions (Figure 6). These findings offer practical implications for poultry health, suggesting that antioxidant intervention may preferentially attenuate apoptosis-associated injury under the present *in-vitro* exposure conditions. However, this study has limitations. First, direct quantification of cadmium adsorption to PS-NPs and intracellular cadmium uptake (e.g., by ICP-MS) were not conducted, and this remains a key direction for future work. Second, the findings are based on an *in vitro* model and require validation in live animals. Third, although our evidence demonstrates the activation of the NLRP3/Caspase-1/GSDMD pathway, we cannot definitively establish this pathway as the exclusive causal mechanism driving pyroptosis due to the lack of specific pharmacological inhibitors (e.g., MCC950) or genetic knockdown experiments. Future studies should explore *in vivo* toxicity and alternative therapeutic targets for pyroptosis.

**Figure 6:**
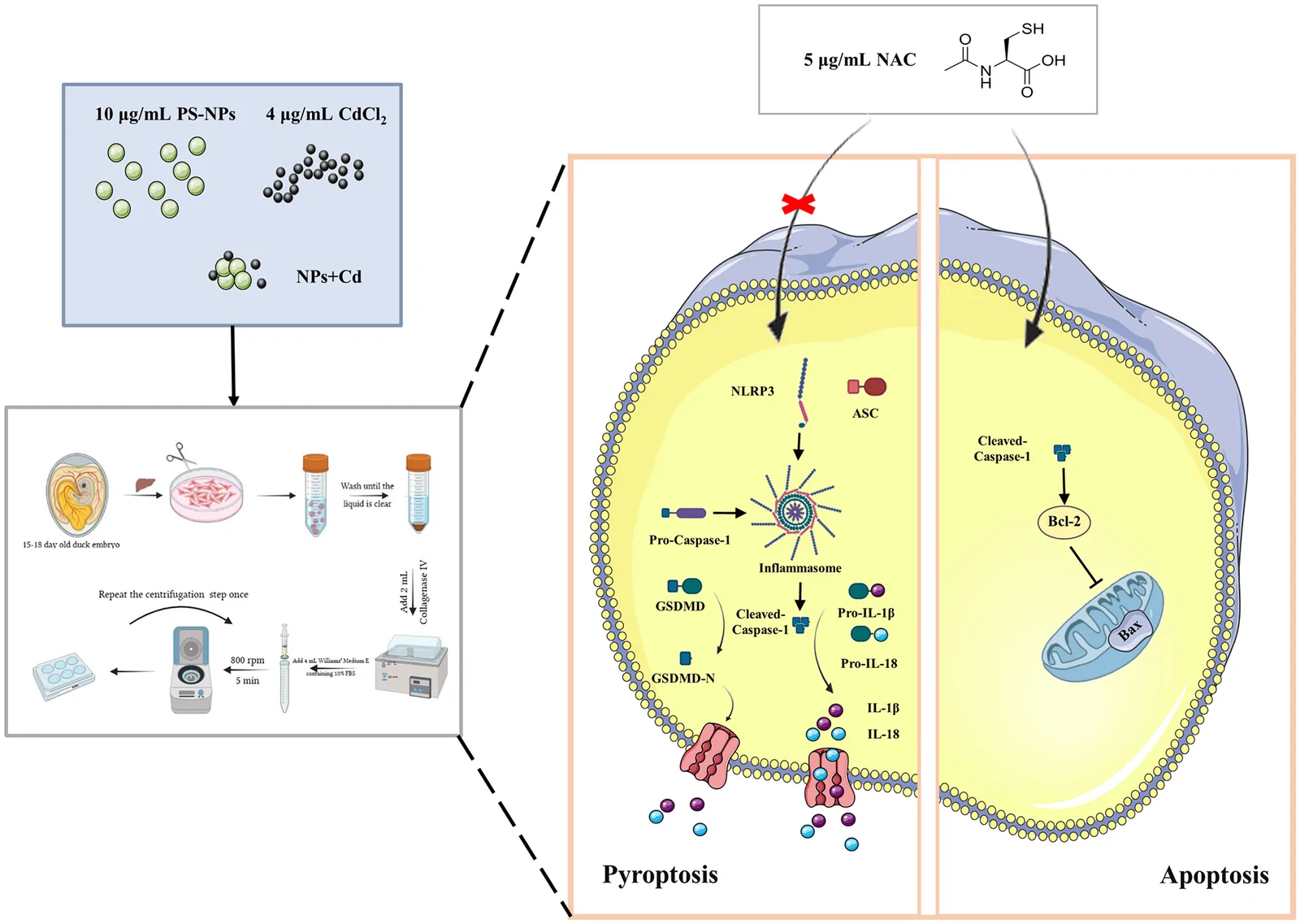
Schematic diagram of the regulatory network for the polystyrene nanoplastics aggravate cadmium-induced pyroptosis and apoptosis in duck hepatocytes. The left panel illustrates the experimental workflow, involving the isolation of primary duck embryo hepatocytes (PDEH) from 15–18-day-old duck embryos and subsequent exposure to polystyrene nanoplastics (PS-NPs, 10 μg/mL) and cadmium chloride (CdCl_2_, 4 μg/mL). The right panel depicts the differential regulatory effects of N-acetylcysteine (NAC, 5 μg/mL). Notably, NAC treatment significantly reversed apoptosis by modulating the Bax/Bcl-2 mitochondrial pathway, but failed to mitigate pyroptosis mediated by the canonical NLRP3/Caspase-1/GSDMD signaling cascade (as indicated by the red cross).

## Data Availability

The raw data supporting the conclusions of this article will be made available by the authors, without undue reservation.
